# Fetal Urinary Ascites From Bladder Rupture: A Rare Complication of Posterior Urethral Valve

**DOI:** 10.7759/cureus.66462

**Published:** 2024-08-08

**Authors:** Asawari Deshmukh, Sanika Deshmukh

**Affiliations:** 1 Radiology and Fetal Medicine, Dhruv Diagnostic and Imaging Clinic, Nagpur, IND; 2 Radiodiagnosis, Dr. D. Y. Patil Medical College, Hospital and Research Centre, Pune, IND

**Keywords:** lower urinary tract obstruction (luto), posterior urethral valve, antenatal sonography, bladder rupture, fetal urinary ascites

## Abstract

Prenatal ultrasonography (USG) plays a crucial role in diagnosing fetal urinary tract anomalies and distinguishing between lower urinary tract obstructive (LUTO) and neurological causes (seen with spinal dysraphism, myelomeningocele, meningocele, and sacral agenesis) of urinary bladder distension. Fetal urinary ascites, a rare but severe complication, can result from bladder rupture associated with obstructive uropathy such as posterior urethral valves (PUV). This case study presents a rare instance of fetal urinary ascites due to PUV detected during prenatal ultrasonography at 20 weeks of gestation (WOG). By highlighting this uncommon but clinically significant condition, we aim to enhance the understanding and management of similar cases in clinical practice.

## Introduction

Posterior urethral valves (PUV) are congenital obstructive membranes in the posterior urethra of male fetuses, forming early in gestation. This obstruction subjects the bladder and upper urinary tract to high pressure, potentially leading to long-term bladder dysfunction and kidney damage. Ultrasonography (USG) is the preferred diagnostic tool for identifying urinary tract abnormalities, including hydroureteronephrosis, bladder wall thickening, and the characteristic "keyhole" sign of PUV [1].

Fetal urinary ascites, although rare, can occur as a complication of PUV due to urinary extravasation following bladder rupture. This condition poses significant risks, including pulmonary hypoplasia and renal dysplasia, which can result in severe perinatal morbidity and mortality. Early and accurate prenatal diagnosis through USG is essential for optimal management and counseling of affected pregnancies.

In this case report, we describe a 20-week gestation fetus with urinary ascites secondary to bladder rupture in the presence of lower urinary tract obstruction (LUTO) in the form of PUV, detected through detailed prenatal ultrasound. This report aims to contribute to the existing knowledge of PUV and its complications, emphasizing the importance of early diagnosis and multidisciplinary management in improving outcomes for similar cases.

## Case presentation

A 20-year-old primigravida in a non-consanguineous marriage was sent to our fetal medicine clinic for an anomaly scan at 20 weeks of gestation (WOG). The mother's blood type was B positive, there was no history of illness, and the pregnancy had progressed normally until then. Previous prenatal nuchal translucency (NT) scan at 12 WOG was unremarkable.

**Figure 1:**
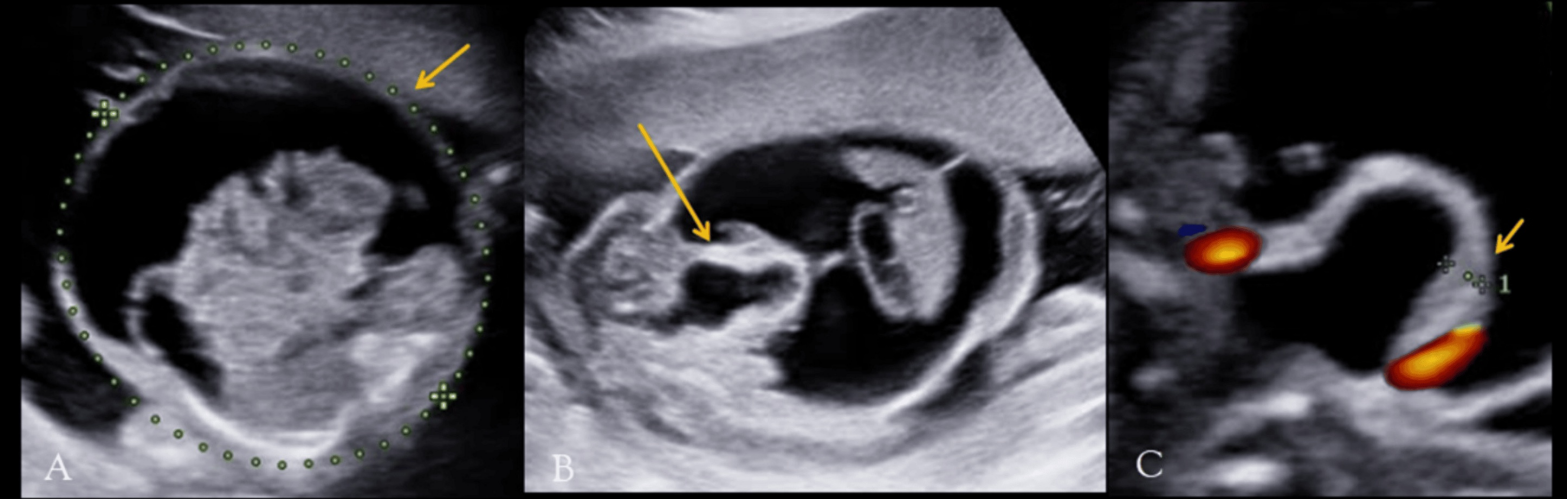
Antenatal ultrasound of the 20-week-old fetus shows increased abdominal circumference measuring 189 mm (dotted line, A), with anechoic fluid collection within the peritoneal cavity (arrow, A). The "keyhole" sign of posterior urethral valve is seen (arrow, B), and the thickened urinary bladder wall is seen (arrow, C), measuring >3.5 mm.

**Figure 2:**
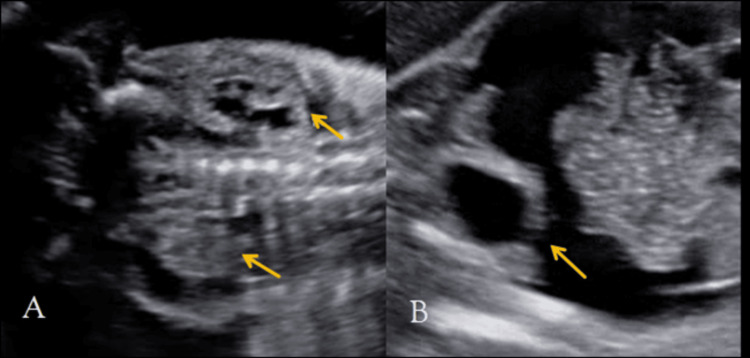
Ultrasound of the upper urinary tract shows bilateral hydronephrosis (arrows, A). A breech in the continuity of the urinary bladder wall is seen (arrow, B), indicating the cause of ascites in a fetus with a posterior urethral valve.

On antenatal ultrasonography, the abdominal circumference of the fetus measured 186 mm (corresponding to 23 + 4 WOG, >95th centile), with significant intraperitoneal anechoic fluid accumulation (Figure 1A). On a dedicated urinary tract scan, the fetus showed posterior urethral valves (keyhole sign) (Figure 1B) and a thickened urinary bladder wall measuring >3.5 mm (Figure 1B), bilateral hydronephrosis (Figure 2A), and a breech in the bladder wall (Figure 2B). Mild oligohydramnios was seen.

Other structures including the bowel, heart, and spine appeared normal on the anomaly scan. Bilateral uterine, and middle cerebral and umbilical artery Doppler showed normal waveform, peak systolic velocity, resistive index, and pulsatility indices, ruling out fetal anemia and pregnancy-induced hypertension (PIH).

The parents decided to abort the fetus after consulting with a radiologist specializing in fetal medicine, pediatric surgeon, and gynecologist. Karyotyping of the abortus was done, which revealed a normal set of chromosomes.

## Discussion

Ultrasonography of the kidneys and bladder is commonly used as an initial imaging test since it does not use ionizing radiation and is readily available, and due to its quick performance. This modality is effective in detecting a range of urinary tract abnormalities, such as hydroureteronephrosis, distension of the bladder, thickening of the bladder wall, and dilated posterior urethra, frequently referred to as the "keyhole" sign [1,2].

During embryogenesis, the caudal end of the Wolffian duct is absorbed into the primordial cloaca at the location of the future seminal vesicle in the posterior urethra [3-5]. This abnormal relative obstructive membrane makes up the posterior urethral valve, which is found inside the posterior urethra in male fetuses.

In a study done by Bernardes et al. [6] to determine the accuracy of the keyhole sign for the accurate diagnosis of PUV in 54 individuals, the prenatal diagnosis of PUV had a sensitivity of 94% and specificity of 43%. Increased bladder wall thickness and dilatation were significantly related to the diagnosis (P < 0.001). However, the presence of the keyhole sign did not indicate a diagnosis of PUV (P = 0.27).

Multiple factors can lead to fetal ascites, including fetal anemia from rhesus immunization, chromosomal abnormalities, TORCH infections (toxoplasmosis, rubella cytomegalovirus, herpes simplex, and HIV), cardiac abnormalities, abdominal or thoracic fetal mass, perforation, meconium peritonitis, and urinary origin (PUV, junction syndrome, cloaca, or idiopathic) [7]. In the indexed case, there was no history of any infection and no evidence of fetal anemia, chromosomal abnormalities, and other structural defects.

Increased pressure inside the fetal urinary system as a consequence of obstructive uropathy, such as posterior urethral valves, can result in urine extravasation [8]. In PUV, transudation across the intact upper tract, ruptured calyceal fornices, or bladder perforation can lead to urinary peritonitis. Most typically, the extravasation appears as urinary ascites and can be found in 25% of fetuses with urethral obstruction [9]. Urinary bladder perforation leading to urinary ascites is a rare complication of PUV [10]. In our case, the urethral obstruction due to PUV leads to upstream dilatation of the bilateral renal pelvis and calyces, and urinary extravasation was seen via the ruptured bladder wall.

PUV induces pulmonary hypoplasia with oligohydramnios in the prenatal stage and has been linked to perinatal mortality and morbidity by disrupting renal development. A careful examination of the fetal spine, bowel, and heart should be performed [11]. Fetal urethral obstructions are related to a variety of chromosomal abnormalities such as trisomies 21 and 18 [12-14]. In our case, on a detailed anomaly scan, the fetal spine, bowel, and heart appeared normal, and karyotyping showed a normal set of chromosomes.

Pinette et al. [15] gave a diagnostic approach for enlarged fetal bladder in which they stated that prenatal sonographic abnormalities in PUV also warranted repeated sonographic monitoring to check for changes in bladder enlargement, persistence of findings, and amniotic fluid volume. They further noted that PUV produces pulmonary hypoplasia with oligohydramnios in the fetal stage and is linked to perinatal mortality and morbidity by disrupting renal development. Oligohydramnios, urinary ascites, perinephric urinoma, increased renal echogenicity, and cortical cysts are indications for poor prognostic indicators. When PUV is suspected before the second trimester, there is a significant risk of end-stage renal disease and substantial perinatal mortality. This risk is especially high if there is severe bilateral hydronephrosis with oligohydramnios and symptoms that point to renal dysplasia [10,16,17]. PUV may not be apparent on prenatal ultrasonography until the second trimester [18]. In the indexed case, the first trimester NT scan at 12 weeks of gestation did not show PUV.

Matsell et al. [19] studied antenatal determinants of long-term kidney outcome in 82 boys with posterior urethral valves and concluded that pulmonary hypoplasia, renal failure, and sepsis were previously the main causes of perinatal mortality, which stood at 50%. Because prenatal diagnosis and perinatal care have improved, this percentage has dropped to less than 3%. The capacity for early detection has been made possible by the use of ultrasonography in fetuses.

In their review of the consensus on the management of posterior urethral valves, Sharma et al. [11] stated that the diagnosis is predicated on sonological findings that ought to be verified by two independent observers, one of whom ought to be a radiologist with training in prenatal anomaly sonography. In India, a woman can lawfully end her pregnancy if the prognosis for her unborn child is poor. Furthermore, sex determination is both illegal and penalized. Therefore, in the event that an obstructive uropathy is detected prior to 20 weeks of gestation, the parents are notified of the anomaly's prognosis. Most young parents who have little obstetric history decide to end their pregnancy medically after speaking with radiologists, pediatric surgeons, and gynecologists [11]. In the indexed case, the parents decided to abort the fetus after consulting with a radiologist specializing in fetal medicine, pediatric surgeon, and gynecologist.

## Conclusions

This case report highlights the critical role of prenatal ultrasound in diagnosing posterior urethral valves (PUV) and associated complications such as fetal urinary ascites. The early identification of PUV through detailed ultrasonographic assessment, including the presence of the keyhole sign, bladder wall thickening, and hydronephrosis, is essential for predicting potential perinatal outcomes. The fetal urinary ascites observed in this case, secondary to bladder rupture, underscores the severe nature of obstructive uropathy. Prompt detection allows for informed decision-making regarding pregnancy management and potential interventions. The decision to terminate the pregnancy was based on a multidisciplinary approach, considering the significant risk of morbidity and mortality associated with severe PUV. This case adds to the existing knowledge of PUV and its complications, emphasizing the importance of thorough prenatal evaluation and interdisciplinary collaboration in managing such complex cases.
